# New Hypoglycemic Drugs: Combination Drugs and Targets Discovery

**DOI:** 10.3389/fphar.2022.877797

**Published:** 2022-06-08

**Authors:** Xiayun Ni, Lei Zhang, Xiaojun Feng, Liqin Tang

**Affiliations:** Department of Pharmacy, The First Affiliated Hospital of University of USTC, Division of Life Sciences and Medicine, University of Science and Technology of China (USTC), Hefei, China

**Keywords:** type 2 diabetes, combination medication, DPP-4i, GLP-1RA, SGLT-2i

## Abstract

New hypoglycemic drugs, including glucagon-like peptide 1 receptor agonists (GLP-1RA), dipeptidyl peptidase-4 inhibitors (DPP-4i) and sodium-glucose cotransporter 2 inhibitors (SGLT-2i), which brings more options for the treatment of type 2 diabetes (T2DM). They are generally well tolerated, although caution is required in rare cases. Clinical trials have show good glycemic control with combination therapy with new hypoglycemic drugs in prediabetes and T2DM (mostly traditional stepwise therapy), but early combination therapy appears to have faster, more, and longer-lasting benefits. With the widespread clinical application of oral semaglutide, it is time to develop combinations drugs containing new hypoglycemic drugs, especially SGLT-2i and/or GLP-1RA, to control the risk of prediabetes and newly diagnosed T2DM and its cardiovascular complications, while improving patient compliance. Clinical and preclinical studies support that SGLT-2i exerts its protective effect on heart failure through indirect and direct effects. How this comprehensive protective effect regulates the dynamic changes of heart genes needs further study. We provide ideas for the development of heart failure drugs from the perspective of “clinical drug-mechanism-intensive disease treatment.” This will help to accelerate the development of heart failure drugs, and to some extent guide the use of heart failure drugs.

## 1 Introduction

According to IDF diabetes atlas 10th edition: Globally, 1 out of every 10 adults aged 20–79 have diabetes. There are a total of 537 million adult patients, of which type 2 diabetes (T2DM) accounts for the vast majority (>90%). It is estimated that by 2030 and 2045, this number will increase to 643 million and 783 million, respectively. In 2021, diabetes caused 6.7 million deaths worldwide (this means 1 person dies of diabetes every 5 s). Chronic high glucose may cause damage to the cardiovascular system, eyes, kidneys, and nerves, which in turn may lead to a series of complications of diabetes. Cardiovascular diseases (CVD), including atherosclerosis and heart failure (HF) are still the main causes of premature death in patients with diabetes (Stumvoll et al., 2005; Rosano et al., 2017). The prevalence of cardiac dysfunction in patients with type 1 diabetes (T1DM) and T2DM is 14.5 and 35.0%, respectively (Konduracka et al., 2013; Bouthoorn et al., 2018). Moreover, the risk of HF in diabetic patients is related to the level of blood glucose: when the glycated haemoglobin (HbA_1c_) level increases by 1%, the risk of HF in patients with T1DM and T2DM increases by 30% and 8%, respectively (Jia et al., 2018).

The important goal of the treatment of T2DM is to maintain blood glucose management and decrease the incidence of T2DM complications (especially CVD) (Rosenstock et al., 2019a). HbA_1c_ is a vital indicator of blood glucose management and diabetes complications (American, 2021). Many non-pregnant adults are suitable for HbA_1c_ control target <7% (53 mmol/mol) and no significant hypoglycemia is recommended. Setting lower HbA_1c_ levels (<7%) may have greater benefits if it can be done safely. Less stringent HbA_1c_ goals (for example, <8%) may apply to patients with limited life expectancy or with treatment that does more harm than good (American, 2021). Metformin has been one of the most popular oral hypoglycemic drugs for the past 60 years, and is still the first-line drug for the initial therapy of most T2DM patients (Sanchez-Rangel and Inzucchi, 2017; Flory and Lipska, 2019; Feng et al., 2021). In recent years, the approval of a large number of new hypoglycemic drugs has brought more options for the therapy of T2DM patients, including DPP-4i, GLP-1RA, and SGLT-2i.

To fully understand the role of new hypoglycemic drugs in T2DM (including prediabetes), it is necessary to conduct a comprehensive study of their antidiabetic capacity, additional benefits and risks, how to use for maximum potential, and their role in new drug development.

## 2 New Hypoglycemic Drugs

### 2.1 Blood Glucose and Weight Management

SGLT-2i have a special hypoglycemic pathway, which does not rely on insulin, but reduces the glucose reabsorption by the proximal renal tubules, resulting in increased excretion of glucose from the urine, thereby controlling the blood glucose of patients with T2DM (Das et al., 20202020; Weng et al., 2021). In the case of hyperglycemia, this inhibitory effect of glucose reabsorption is more obvious (Riddle, 2010). GLP-1 receptor exists in a variety of tissues and cells, including brain, kidney, cardiomyocytes, and vascular endothelial cells (Ban et al., 2008). When blood glucose level is high, GLP-1RA activate GLP-1 receptor to stimulate pancreatic β cells to secrete insulin while reduce the secretion of glucagon from pancreatic α cells, thereby improving blood glucose levels. When blood glucose level is low, GLP-1RA have no stimulating effect on insulin secretion, which helps decrease the risk of hypoglycemia (Farilla et al., 2002; Sfairopoulos et al., 2018; Sharma et al., 2018; Sposito et al., 2018). 2021 ESC Guidelines on heart failure management list SGLT2i as one of the main treatment drugs for patients with HF (Authors/Task Force et al., 20212022). ADA Standards of Medical Care in Diabetes (2022): For patients with atherosclerotic cardiovascular disease (ASCVD) or indications of high risk, HF, and chronic kidney disease (CKD), the ADA recommends a regimen containing SGLT-2i (for ASCVD/HF/CKD) or GLP-1RA (for ASCVD) (American Diabetes Association Professional Practice et al., 2022). Considering the overlapping hypoglycemic mechanisms, it is recommended to discontinue DPP-4i when T2DM patients are intensified from DPP-4i to GLP-1 RA (American Diabetes Association Professional Practice et al., 2022).

We list combination therapy trials involving new hypoglycemic drugs (mostly traditional stepwise therapy) in Supplementary Table S1 and Table 1, which are summarized in terms of HbA_1c_ and weight management. There are many clinical studies of GLP-1RA in T2DM, and the efficacy of different GLP-1RA is different (Supplementary Table S1). The current evidence suggests that 1.8 mg liraglutide once-daily is better than exenatide [2 mg once-weekly (Buse et al., 2013) or 10 μg twice-daily (Buse et al., 2009)], lixisenatide 20 μg once-daily (Nauck et al., 2016) and albiglutide 50 mg once-weekly (Pratley et al., 2014), and the effect is equivalent to dulaglutide 1.5 mg once-weekly (Dungan et al., 2014), but weaker than semaglutide (oral) 14 mg once-daily (Pratley et al., 2019). In addition, semaglutide 1.0 mg once per week is better than semaglutide (oral) 2.5–20 mg once-daily (Davies et al., 2017), dulaglutide 1.5 mg once-weekly (Pratley et al., 2018), and exenatide 2 mg once-weekly (Ahmann et al., 2018), but weaker than tirzepatide [a glucose-dependent insulinotropic polypeptide (GIP) and GLP-1 receptor agonist] 5–15 mg once-weekly (Frías et al., 2021a). Further, dulaglutide 1.5 mg once per week is superior to exenatide 10 μg twice-daily (Wysham et al., 2014), and dulaglutide 0.75 mg once per week is weaker than semaglutide (oral) 7 mg once-daily (Yabe et al., 2020). Finally, exenatide 2 mg once per week is better than exenatide 10 μg twice-daily (Wysham et al., 2014) and lixisenatide 20 μg once-daily (Rosenstock et al., 2013).

**TABLE 1:** Randomized controlled trials of GLP-1RA, SGLT-2i and/or DDP-4i in T2DM (mainly phase 3).

	Groups	Duration (wks)	n	Inclusion Criteria	HbA1c changes (%)	Weight Changes (kg)
**GLP-1RA vs. DPP-4i**						
PIONEER 3 Rosenstock et al. (2019b) 2019	Semaglutide 3, 7, 14 mg oral vs. Sitagliptin 100 mg, all once daily	78	1864	T2DM, aged ≥18 years, HbA1c 7.0–10.5%, receiving a stable metformin treatment (with or without sulfonylurea)	−0.6, −1.0, −1.3 vs. −0.8 (26 weeks)	−1.2, −2.2, −3.1 vs. −0.6 (26 weeks)
				Baseline HbA1c (%): 8.3–8.4	HbA1c<7.0%: 27%, 37%, 44% vs. 29%	
				Baseline BMI (kg/m^2^): 32.3–32.6		
PIONEER 7 Pieber et al. (2019) 2019	Semaglutide 3.7 or 14 mg/d (oral, dose flexible) vs. Sitagliptin 100 mg/d	52	504	T2DM, aged ≥18 years, HbA1c 7.5–9.5%, taking stable daily doses of 1–2 OAM (for ≥90 days)	−1.3 vs. −0.8	−2.6 vs. −0.7
				Baseline HbA1c (%): 8.3 (0.6)	HbA1c < 7.0%: 58% vs. 25%	
				Baseline BMI (kg/m2): 31.5 (6.1–6.5)		
DURATION-2 Bergenstal et al. (2010) 2010	Exenatide 2 mg once per week vs. Sitagliptin 100 mg/d vs. pioglitazone 45 mg/d	26	491	T2DM, aged ≥18 years, HbA1c 7.1–11.0%, receiving a stable dosage of metformin	HbA1c < 9.0% change from baseline: −1.1 vs.−0.5 vs.−0.9	−2.3 vs. −0.8 vs. 2.8
				Baseline HbA1c (%): 8.5–8.6	HbA1c ≥ 9.0% change from baseline: −2.0 vs. −1.3 vs. −1.5	
				Baseline BMI (kg/m^2^): 32 (5–6)	HbA1c<7.0%: 60% vs. 35% vs. 52%	
1860-LIRA-DPP-4 Pratley et al. (2010) 2010	Liraglutide 1.2 mg, 1.8 mg vs. Sitagliptin 100 mg, all once daily	26	665	T2DM, aged 18–80 years	−1.24, −1.50 vs. −0.90	−2.86, −3.38 vs. −0.96
				HbA1c 7.5–10.0%, receiving a stable daily dose of metformin (≥1.5 g) for ≥90 days	HbA1c < 7.0% (approximately): 44%, 55% vs. 22%	
				Baseline HbA1c (%): 8.4–8.5		
				Baseline BMI (kg/m2): 32.6–33.1		
**GLP-1RA vs. SGLT-2i**						
SUSTAIN 8 Lingvay et al. (2019) 2019	Semaglutide 1mg, once weekly vs. Canagliflozin 300mg, once daily	52	788	T2DM, aged ≥18 years, HbA1c 7.0–10.5%, recieving a stable metformin treatment (≥1.5 g/d or MTD)	−1.5 vs. −1.0	−5.3 vs. −4.2
				Baseline HbA1c (%): 8.3 (1.0)	HbA1c < 7.0%: 66% vs. 45%	Weight loss ≥5%: 51.1 vs. 46.6%
				Baseline BMI (kg/m2): 32.3 (6.8)		
PIONEER 2 Rodbard and Rosenstock, (2019) 2019	Semaglutide 14 mg/d, oral vs. Empagliflozin 25 mg/d	52	822	T2DM, aged ≥18 years, HbA1c 7.0–10.5%, receiving a stable metformin treatment (≥1.5 g/d or MTD)	−1.3 vs. −0.8	−4.7 vs. −3.8
				Baseline HbA1c (%): 8.1 (0.9)	HbA1c<7.0%: 63.0% vs. 44.0%	
				Baseline BMI(kg/m^2^): 32.8 (6.1)		
**GLP-1RA + SGLT-2i (Simultaneous or sequential use)**						
DURATION-8 Jabbour et al. (2020) 2020	Exenatide 2 mg once weekly + Dapagliflozin 10 mg/d vs. Exenatide 2 mg once weekly, vs. Dapagliflozin 10 mg/d	104	695	T2DM, aged ≥18 years, HbA1c 8.0–12.0%, with stable daily dose of metformin monotherapy (≥1.5 g)	−1.70 vs. −1.29 vs. −1.06	−2.48 vs. −0.77 vs. −2.99
				Baseline HbA1c (%): 9.3 (1.0–1.1)	HbA1c < 7.0% (approximately): 30% vs. 22% vs. 13%	Weight loss ≥5% (approximately): 24% vs. 12% vs. 21%
				Baseline BMI (kg/m^2^): 32.0–33.2		
SUSTAIN 9 Zinman et al. (2019b) 2019	Semaglutide 1.0 mg once per week vs. Placebo	30	302	T2DM, aged ≥18 years, HbA1c 7.0–10.0%, treatment with an SGLT-2i (or with a sulphonylurea or metformin (≥1.5 g/d or MTD) for ≥90 days	−1.5 vs. −0.1	−4.7 vs. −0.9
				Baseline HbA1c (%): 8.0 (0.8)	HbA1c < 7.0%: 78.7% vs. 18.7%	Weight loss ≥5%: 49.9% vs. 8.2%
				Baseline BMI (kg/m^2^): 31.9 (6.6)		
AWARD-10 Ludvik et al. (2018) 2018	Dulaglutide 1.5 mg, 0.75 mg, once per week vs. Placebo	24	422	T2DM, aged ≥18 years, HbA1c 7.0–9.5%, receiving stable doses (>90 days) of an SGLT-2i (with or without metformin)	−1.34, −1.21 vs. −0.54	-3.1, -2.6 vs. -2.1
				Baseline HbA1c (%): 8.04–8.05	HbA1c<7.0%: 71%, 60% vs. 32%	
				Baseline BMI (kg/m2): 32.39–32.87		
**SGLT-2i or GLP-1RA or SGLT-2i + DPP-4i vs. glimepiride or insulin**						
NCT02551874 Vilsbøll et al. (2019) 2019	Dapagliflozin (SGLT- 2i) 10 mg/d + Saxagliptin (DPP-4i) 5 mg/d vs. titrated insulin glargine	24	643	T2DM, aged 18 years or older, HbA1c 8.0–12.0%, receiving a stable daily dose of metformin (≥1.5 g), with or without a stable dose of sulfonylurea (≥50% maximum dose)	−1.7 vs. -1.5	−1.50 vs. 2.14
				Baseline HbA1c (%): 9.1 (1.0)	HbA1c<7.0%: 33.2% vs. 33.5%	
				Baseline BMI (kg/m^2^): 32.2 (5.3)		
EMPA-REG H2H-SU Ridderstråle et al. (2018) 2018	Empagliflozin 25 mg vs. Glimepiride 1–4 mg	208	1,545	T2DM, aged ≥18 years, HbA1c 7.0–10.0%, receiving a stable daily dose of metformin (≥1.5 g)	−0.29 vs. −0.10	Difference: −4.92
				Baseline HbA1c (%): 7.83–7.87	Confirmed hypoglycemia: 3% vs. 28%	
				Baseline BMI (kg/m^2^): 30.29–30.49		
SUSTAIN 4 Aroda et al. (2017) 2017	Semaglutide 0.5, 1.0 mg once per week vs. Insulin glargine (starting dose 10 IU/d)	30	1,089	T2DM, aged ≥18 years, HbA1c 7.0–10.0%, insulin-naive and on therapy with metformin (or metformin + sulfonylurea) for ≥90 days	−1.21, −1.64 vs. −0.83	−3.47, −5.17 vs. 1.15
				Baseline HbA1c (%): 8.2 (0.9)	HbA1c < 7.0%: 57%, 73% vs. 38%	
				Baseline BMI (kg/m^2^): 33.0 (6.5)		

Abbreviations: ADA, the American Diabetes Association; BMI, body-mass index; DPP-4i, dipeptidyl peptidase-4, inhibitors; GLP-1RA, glucagon-like peptide 1 receptor agonists; HbA1c, glycated haemoglobin; MTD, maximum tolerated dose; OAM, oral antihyperglycemic medication; SGLT-2i, sodium-glucose cotransporter 2 inhibitors.

Table 1 lists blood glucose and weight management differences in other cases, including: GLP-1RA vs. DPP-4i, GLP-1RA vs. SGLT-2i, and GLP-1RA + SGLT-2i (Simultaneous or sequential use), and SGLT-2i or GLP-1RA or SGLT-2i + DPP-4i vs. glimepiride or insulin. The main advantages of new hypoglycemic drugs are their significant hypoglycemic effect and low incidence of hypoglycemic adverse reactions, especially when compared with insulin and sulfonylureas. In addition, SGLT2i and GLP-1RA have obvious cardio-renal protective effects, and DPP-4i has advantages in patients who do not need to lose weight.

### 2.2 Benefits Beyond the Hypoglycemic Effect of New Hypoglycemic Drugs

#### 2.2.1 Protection of Heart and Kidney Function

Recently, the meta-analysis of medium to high certainty evidence by Kanie et al. (2021) showed that: GLP-1RA and SGLT-2i may decrease the CVD mortality and all-cause mortality in patients with confirmed CVD; Moderately certain evidence may support that SGLT-2i reduces the incidence of worsening renal function, while DPP-4i treatment does not affect CVD mortality and all-cause mortality; SGLT-2i ranks best in reducing CVD and all-cause mortality; High-certainty evidence suggests that SGLT-2i treatment decreases the risk of hospitalization due to HF, and moderate-certainty evidence may support GLP-1RA treatment to reduce fatal and non-fatal strokes.

Unlike SGLT-2i, GLP-1RA does not have a clinical study with kidney as the main outcome. The ongoing FLOW trial has studied the effects of semaglutide (injection) on the renal outcome of T2DM patients with CKD, and will provide further treatment and mechanism insights (NCT03819153). The effect of GLP-1RA on the progression of CKD needs to be further studied (Brown et al., 2021).

In fact, in patients with non-T2DM, SGLT-2i, and GLP-1RA also have convincing data to support their cardiac benefits. For example, the findings of the DAPA-HF study showed that dapagliflozin lowered the risk of worsening HF and death due to cardiovascular events in non-diabetic patients with HF (reduced ejection fraction, HFrEF) (HR 0.74; 0.65–0.85 and 0.82; 0.69–0.98, respectively) (McMurray et al., 2019). Similarly, the results of the EMPEROR-Reduced study showed that empagliflozin reduced the risk of hospitalization or death due to HF in the empagliflozin group was lower, regardless of whether diabetes was present or not (HR 0.75; 0.65–0.86) (Packer et al., 2020).

There has been little progress in the treatment of patients with HF (preserved ejection fraction, HFpEF), and SGLT-2i has broad prospects for the therapy of HFpEF. For example, the results of the EMPEROR-Preserved study indicated that empagliflozin decreased the risk of hospitalization or death due to worsening HF (HR 0.79; 0.69–0.90) (Anker et al., 2021). Similarly, dapagliflozin treatment for 12 weeks obviously improved the symptoms, motor function, and physical limitations reported by patients with chronic HFpEF, and it was well tolerated (NCT03030235) (Nassif et al., 2021). The beneficial effects of empagliflozin and dapagliflozin are also not related to diabetes status (Anker et al., 2021; Nassif et al., 2021). In addition, in the SOLOIST-WHF study (all patients have T2DM), the benefits of sotagliflozin in patients with HFrEF and HFpEF are consistent (Bhatt et al., 2021). The ongoing DELIVER trial (NCT03619213) will further determine the effect of dapagliflozin added to the routine therapy of patients with HF (reserved or slightly reduced ejection fraction) (Solomon et al., 2021). We list the effects of SGLT-2i and GLP-1RA on cardiac and/or renal function in patients with T2DM in Table 2.

**TABLE 2:** The effect of SGLT-2i on cardiorenal outcome and GLP-1RA on cardiovascular outcome.

	Groups	*n*	Duration (years)	Inclusion Criteria	Key Cardiovascular Outcome	Key Renal Outcome
**Effect of SGLT-2i On Cardiorenal Outcomes in T2DM**						
EMPA-REG Zinman et al. (2015) 2015	Empagliflozin (10 or 25 mg/d) vs. Placebo	7,020	Median observation time: 3.1	eGFR >30 ml/min/1.73 m^2^, established CVD	Death: 12.4 vs. 20.2	Key renal outcome
					Hospital for HF: 9.4 vs. 14.5	
					All-cause mortality: 19.4 vs. 28.6	
					Myocardial infarction: 16.8 vs. 19.3	
					Stroke: 12.3 vs. 10.5	
					Adverse events: 37.4 vs. 43.9	
DECLARE-TIMI 58 Wiviott et al. (2019) 2019	Dapagliflozin 10 mg/d vs. Placebo	17,160	Median observation time: 4.2	Multiple ASCVD risk factors (59.4%) or established CVD (40.6%)	Death: 7.0 vs. 7.1	>40% decrease to eGFR <60 ml/min/1.73 m^2^, ESKD, or renal/CV death: 3.7 vs. 7.0
					Hospital for HF: 6.2 vs. 8.5	
					All-cause mortality: 15.1 vs. 16.4	
					Myocardial infarction: 11.7 vs. 13.2	
					Stroke: 6.9 vs. 6.8	
					Adverse events: 22.6 vs. 24.2	
CANVAS Neal et al. (2017a) 2017	Canagliflozin 300 mg/d vs. placebo	10,142	Median observation time: 2.62	Established CVD	Death: 11.6 vs. 12.8	40% decrease in eGFR, renal replacement treatment, or renal-related death: 5.5 vs. 9.0 Albuminuria: 89.4 vs. 128.7
					Hospital for HF: 5.5 vs. 8.7	
					All-cause mortality: 17.3 vs. 19.5	
					Myocardial infarction: 11.2 vs. 12.6	
					Stroke: 7.9 vs. 9.6	
					Adverse events 26.9 vs. 31.5	
VERTIS Cannon et al. (2020)2020	Ertugliflozin (5 or 15 mg/d) vs. Placebo	8,246	Followed for a mean: 3.5	Aged >40 years, established CVD	Death: 6.2 vs. 6.7	
					Hospital for HF: 2.5 vs. 3.6	
					All-cause mortality: 8.6 vs. 9.2	
					Myocardial infarction: 6.0 vs. 5.8	
					Stroke: 3.4 vs. 3.2	
					Adverse events: 11.9 vs. 11.9	
CREDENCE Perkovic et al. (2019a) 2019	Canagliflozin 100 mg/d vs. Placebo	4,401	Median observation time: 2.62	AlbuminuricCKD: eGFR 30–90 ml/min/1.73 m^2^and an albumin-to-creatinine ratio of 300–5,000 mg/g	Death: 19.0 vs. 24.4	Serum creatinine doubled, ESKD, or renal/CV death: 43.2 vs. 61.2
					Hospital for HF: 15.7 vs. 25.3	Dialysis, renal replacement treatment, or renal related death: 13.6 vs. 18.6
					All-cause mortality: 29.0 vs. 35.0	
					Adverse events: 38.7 vs. 48.7	ESKD: 20.4 vs. 29.4
SOLOIST-WHF Bhatt et al. (2021) 2021	Sotagliflozin 200–400 mg/d vs. Placebo	1,222	Followed for a mean: 9.0 months	eGFR >30 ml/min/1.73 m^2^, Recently hospitalized for worsening HF	Death: 10.6 vs. 12.5	Acute kidney injury: 4.1 vs. 4.4
					Hospital and urgent visits for HF: 40.4 vs. 63.9	
					All-cause mortality: 13.5 vs. 16.3	Any renal or urinary disorders: 11.6 vs. 12.3
					Adverse events: 69.4 vs. 67.4	
**Effect of GLP-1RA on cardiovascular outcomes in T2DM**						
ELIXA (Pfeffer et al., 2015) 2015	Lixisenatide 20 μg/d vs. Placebo	6,068	Followed for a mean: 2.08	Acute coronary event <180 days before screening	MACE: 13.4 vs. 13.2	Hospital for HF: 4.0 vs. 4.2
					Death: 5.1 vs. 5.2	Myocardial infarction: 8.9 vs. 8.6
					All-cause mortality: 7.0 vs. 7.4	Stroke: 2.2 vs. 2.0
LEADER Marso et al. (2016a) 2016	Liraglutide 1.8 mg/d vs. Placebo	9,340	Median observation time: 3.8	Age >50 years with established CVD, CKD, or HF or age >60 years with ≥1 known risk factor	MACE: 13.0 vs. 14.9	Hospital for HF: 4.7 vs. 5.3
					Death: 4.7 vs. 6.0	Myocardial infarction: 6.3 vs. 7.3
					All-cause mortality: 8.2 vs. 9.6	Stroke: 3.7 vs. 4.3
SUSTAIN-6 (Marso et al., 2016b) 2016	Semaglutide (0.5 or 1.0 mg) once per week vs. Placebo	3,297		Age >50 years with established CVD, CKD, or HF or age >60 years with ≥1 known risk factor	MACE: 6.6 vs. 8.9	Hospital for HF: 3.6 vs. 3.3
					Death: 2.7 vs. 2.8	Myocardial infarction: 2.9 vs. 3.9
					All-cause mortality: 3.8 vs. 3.6	Stroke: 1.6 vs. 2.7
EXSCEL (Holman et al., 2017) 2017	Exenatide 2 mg once weekly vs. Placebo	14,752	Median observation time: 3.2	Established CVD (73.1%) or multiple CV risk factors	MACE: 11.4 vs. 12.2	Hospital for HF: 3.0 vs. 3.1
					Death: 4.6 vs. 5.2	Myocardial infarction: 6.6 vs. 6.7
					All-cause mortality: 6.9 vs. 7.9	Stroke: 2.5 vs. 2.9
HARMONY (Hernandez et al., 2018) 2018	Albiglutide 30∼50 mg once weekly vs. Placebo	9,463	Median observation time: 1.6	Age >40 years and ASCVD	MACE: 7.0 vs. 9.0	Myocardial infarction: 4.0 vs. 5.0
					Death: 3.0 vs. 3.0	Stroke: 2.0 vs. 2.0
					All-cause mortality: 4.0 vs. 4.0	
REWIND (Gerstein et al., 2019) 2019	Dulaglutide 1.5 mg once per week vs. Placebo	9,901	Median observation time: 5.4	Age >50 years, previous CV events, CVD (31.5%) or multiple CV risk factors	MACE: 12.0 vs. 13.4	Hospital for HF: 4.3 vs. 4.6
					Death: 6.4 vs. 7.0	Myocardial infarction: 4.5 vs. 4.7
					All-cause mortality: 10.8 vs. 12.0	Stroke: 3.2 vs. 4.1
PIONEER-6 (Husain et al., 2019) 2019	Semaglutide 14 mg once-daily [oral] vs. Placebo	3,183	Median observation time: 1.325	Age >50 years with CVD (84.7%) or age >60 years with ≥1 CV risk factor	MACE: 3.8 vs. 4.8	Hospital for HF: 1.3 vs. 1.5
					Death: 0.9 vs. 1.9	Myocardial infarction: 2.3 vs. 1.9
					All-cause mortality: 1.4 vs. 2.8	Stroke: 0.8 vs. 1.0

Abbreviations: ASCVD, atherosclerotic cardiovascular disease; CKD, chronic kidney disease; CV, cardiovascular; CVD, cardiovascular disease; eGFR, estimated glomerular filtration rate; ESKD, end-stage kidney disease; HF, heart failure; MACE, major adverse cardiovascular events.

#### 2.2.2 Weight and Blood Pressure

**FIGURE 1:**
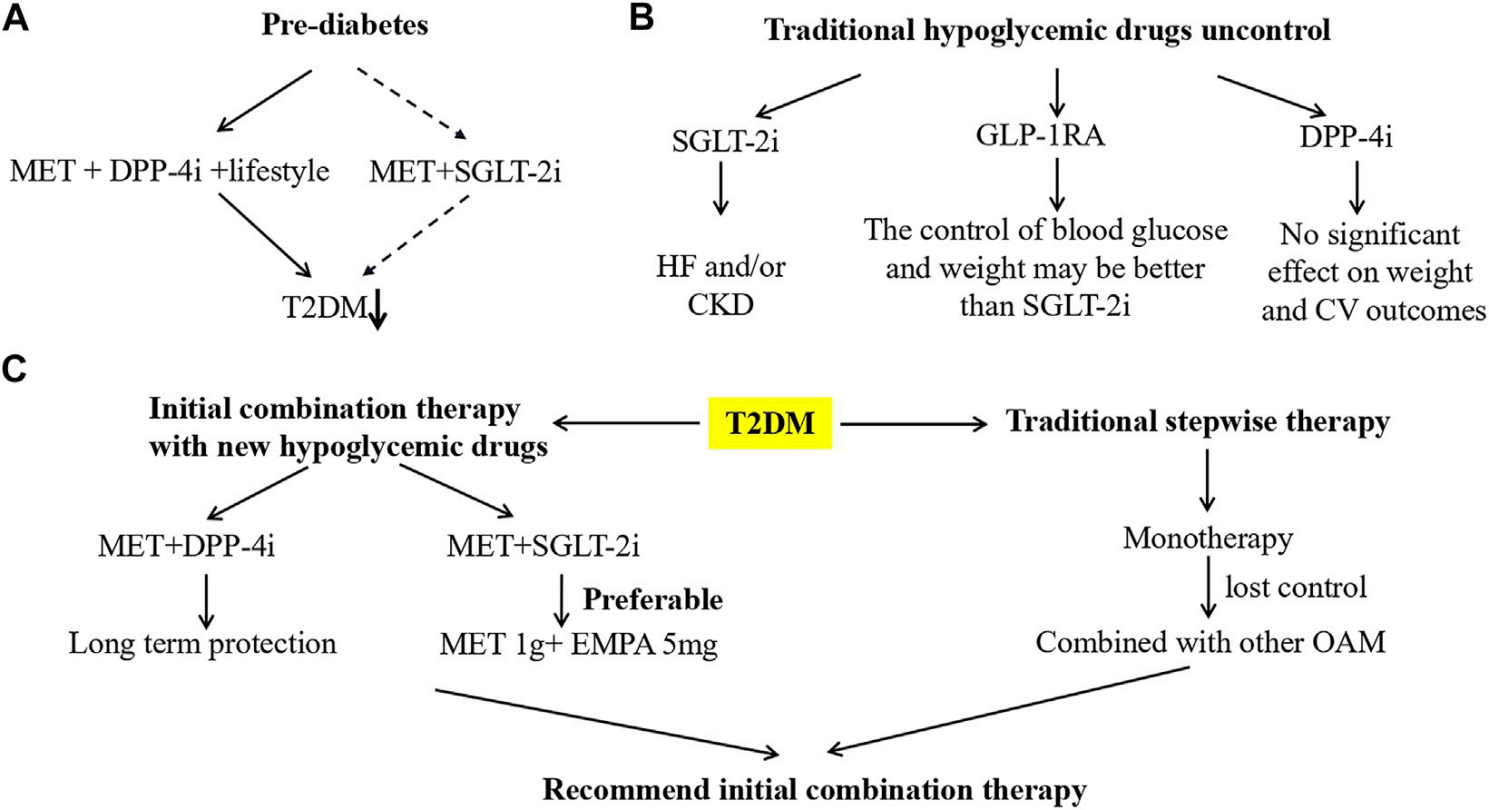
Combined use of hypoglycemic drugs in different situations. **(A)** Pre-diabetes is a very common health problem, and the risk of progression to T2DM and/or diabetes complications is high. But given the reduced morbidity in the short term, and the modest benefit in terms of side effects and/or lack of proven cardiovascular benefit. On this basis, the FDA and EMA in the United States and Europe announced that there are no drugs recommended for T2DM prevention. Nevertheless, DPP-4i (linagliptin) + MET + lifestyle may be a valuable option for reducing the risk of patients with prediabetes. In addition, for SGLT-2i (such as dapagliflozin) combined with MET may have similar or better effects (because metformin and dapagliflozin can decrease the risk of new-onset diabetes by 31 and 32%, respectively). **(B)** For T2DM patients who have been using hypoglycemic drugs, when the traditional treatment drugs are not effective, SGLT-2i and/or GLP-1RA and/or DPP-4i can be further added for treatment. For T2DM patients with HF and/or CKD, SGLT2i is preferred. GLP-1RA may be better than SGLT2i in controlling blood glucose and body weight, (for differences between different GLP-1RAs, please see the second paragraph of 2.1), while DPP-4i has no significant effect on body weight and CV outcome. **(C)** For T2DM patients who have not previously used hypoglycemic drugs, compared with traditional stepwise therapy, the initial combination of MET + DPP-4i provides long-term benefits. In addition, compared with MET or SGLT-2i monotherapy, the initial MET + SGLT-2i combination therapy has significant advantages in controlling blood glucose and body weight. Moreover, MET 1 g + EMPA 5 mg (both twice daily) combined therapy is superior to empagliflozin 25 mg/d or metformin 1 g twice daily in controlling blood glucose and weight. Abbreviations: CKD, chronic kidney disease; CV, cardiovascular; DPP-4i, dipeptidyl peptidase-4 inhibitors; EMPA, empagliflozin; GLP-1RA, glucagon-like peptide 1 receptor agonists; HF, heart failure; Met, metformin; OAM, oral antihyperglycemic medication; SGLT-2i, sodium-glucose cotransporter 2 inhibitors; T2DM, type 2 diabetes.

Liraglutide (GLP-1RA) has been approved for weight management in many countries (Brown et al., 2021). Similarly, semaglutide is also being assessed as a drug for the therapy of obesity (Figure 1B). For example, among 1961 non-diabetic adult overweight or obese participants, after 68 weeks of treatment, the weight change of 2.4 mg semaglutide + lifestyle from baseline was −15.3 kg, and the weight change of placebo + lifestyle was −2.6 kg. In addition, subjects in the semaglutide group had greater improvements in cardiovascular and metabolic risk factors (Wilding et al., 2021). SGLT-2i can reduce weight (approximately 2 kg), systolic blood pressure (SBP) (approximately 2.5–5.0 mmHg), and diastolic blood pressure (DBP) (approximately 1–2 mmHg), which has clinical significance (Brown et al., 2021). Similarly, GLP-1RA have shown consistent efficacy in blood pressure (2–3 mmHg) (Brown et al., 2021). These effects of improving cardiovascular metabolic risk factors provide a theoretical basis for GLP-1RA treatment in patients with CV risk factors or CVD.

### 2.3 Risks Beyond the Hypoglycemic Effect of New Hypoglycemic Drugs

#### 2.3.1 Hypoglycemia and Fractures

The meta-analysis of Kanie et al. (2021) showed that: moderately certain evidence may support that SGLT-2i and DPP-4i have no significant effect on hypoglycemia and bone fracture, and it is uncertain that GLP-1RA has an effect on hypoglycemia and bone fracture. In fact, GLP-1RA is a type of incretin, which plays a hypoglycemic effect by increasing the insulin secretion of pancreatic β cells stimulated by glucose (Farilla et al., 2002). Insulin secretion only increases when glucose is higher than about 3.5 mmol/L. Because GLP-1RA action is glucose-dependent, they have a low risk of hypoglycemia, unless combined with sulfonylureas or insulin (Brown et al., 2021). Similarly, adding DPP-4i to sulfonylureas to treat T2DM increased the risk of hypoglycemia by 50% during the first 6 months of treatment, and one case of excessive hypoglycemia occurred in every 17 patients. This highlights the need to follow the recommendations for reducing the dose of sulfonylurea drugs when starting DPP-4i (Salvo et al., 2016).

#### 2.3.2 Genital Infections and Urinary Tract Infections

canagliflozin, empagliflozin, and dapagliflozin have a higher risk of genital infections (ORs 3.21–5.23). Among these SGLT-2i, only dapagliflozin was associated with an obviously higher Urinary Tract Infections (UTIs) risk compared with other active treatments or placebo (OR 1.28; 1.02–1.61). Compared with dapagliflozin, empagliflozin was associated with a significant decrease in UTIs (OR 0.79; 0.64–0.97) (Li et al., 2017). The genital infections and UTIs caused by SGLT-2i mainly occurred in the 24–26 weeks of treatment (Li et al., 2017). Compared with men, women have obviously higher risks of genital infections and UTIs, regardless of whether SGLT-2i is used or not (Li et al., 2017). Observational studies and meta-analysis have indicated that SGLT-2i do not increase the risk of bacterial UTIs, including pyelonephritis (Li et al., 2017; Dave et al., 2019).

#### 2.3.3 Amputation

In the CANVAS and CANVAS-R study, compared with placebo, patients treated with canagliflozin at higher risk of amputation (HR 1.97; 1.41–2.75), mainly at the metatarsal or toe level (Neal et al., 2017b). Similarly, T2DM patients with CVD who started hypoglycemic therapy in the United States Department of Defense military health system were followed up for a median of 1.6 years (*n* = 25,258). The results showed that the use of SGLT-2i increased the risk of lower knee amputation (HR 1.99; 1.12–3.51). The findings highlight the potential benefits and risks that need to be considered when launching SGLT-2i (Udell et al., 2018).

However, canagliflozin increased the risk of amputation and was not found in other subsequent CVOT or CREDENCE (Perkovic et al., 2019b). In addition, Paul et al. (2021) used centricity electronic medical records from the United States to identify 3,293,983 T2DM patients. The results showed that compared with GLP 1-RA, DPP-4i or other anti-diabetic drugs (ADD), the risk of lower limb amputations (LLAs) in SGLT2i is not higher or even lower [HR are: (0.88; 0.73–1.05), (0.65; 0.56–0.75), and (0.43; 0.37–0.49), respectively]. And there was no obvious difference in the incidence of LLA between empagliflozin, canagliflozin, or dapagliflozin. Patients with pre-existing peripheral arterial disease (PAD) had the highest risk (more than fourfold) of developing LLA. These results indicate that SGLT-2i do not increase amputation risk (Paul et al., 2021). Does this mean that PAD patients should be more cautious when using SGLT-2i?

The findings of Bonaca et al. (2020) responded to this question: in patients with T2DM with or without PAD, the use of dapagliflozin has consistent benefits for CVD and kidney disease. No significant difference between placebo and dapagliflozin in any limb outcome (including limb ischemic adverse events and amputation). These results indicate that dapagliflozin may be safe in T2DM patients with PAD (in terms of limb outcome) (Bonaca et al., 2020). Nonetheless, when using SGLT2i, carefully monitor the lower extremity ulcers. If the patient develops lower extremity complications, consider stopping treatment (at least suspend use until symptoms are relieved).

#### 2.3.4 Gastrointestinal Reactions

Gastrointestinal reactions [especially nausea (25–60%) and vomiting (5–15%)] are the most common adverse reactions of GLP-1RA, gradually decreasing over time, usually mild to moderate, the discontinuation rate of gastrointestinal discomfort is lower (5–10%) (Brown et al., 2021). Sometimes, injection site reactions, headaches, and nasopharyngitis may occur (Brown et al., 2021). The weekly preparation of GLP-1RA seems to be more advantageous in lowering glucose and reducing gastrointestinal discomfort (Nauck and Meier, 2019).

#### 2.3.5 Medullary Thyroid Carcinoma, Acute Pancreatitis and Pancreatic Cancer

The C-cell proliferation and medullary thyroid carcinogenesis observed in rodents has not been clinically demonstrated (this may be due to low or absent GLP-1R expression in normal human thyroid tissue). Nevertheless, GLP-1RA should not be recommended when patients at risk of developing medullary thyroid cancer (Brown et al., 2021). GLP-1RA treatment may increase the risk of gallbladder and bile duct disease, and it is more likely to undergo cholecystectomy (Faillie et al., 2016). The meta-analysis of Kanie et al. (2021) indicate that moderately certain evidence may support that DPP-4i increases the risk of pancreatitis (OR 1.63; 1.12–2.37). The good news is that GLP-1RA treatment does not appear to increase the risk of pancreatic cancer or acute pancreatitis compared to other ADD treatments (Storgaard et al., 2017).

#### 2.3.6 Ketoacidosis

It has been reported that diabetic ketoacidosis occurs in patients with T2DM who use GLP-1RA and insulin at the same time when the simultaneous use of insulin is rapidly reduced or stopped. Insulin should be lowered cautiously and gradually, and capillary blood glucose should be monitored (GLP-1 receptor agonists, 2019). Diabetic ketoacidosis of SGLT-2i has also become a spontaneously reported rare adverse reaction (<0.1%), which was subsequently discovered by several large randomized controlled trials, especially in T1DM patients. Risk factors include concurrent use of insulin, concurrent diseases, and major elective surgery or emergency (Brown et al., 2021).

#### 2.3.7 Diabetic Retinopathy

Semaglutide (SUSTAIN-6) was related to a higher risk of diabetic retinopathy compared with placebo (3% vs. 1.8%) (Marso et al., 2016c). Although the rapid glycemic control effect of semaglutide may be responsible for the temporary deterioration of diabetic eye disease, the direct effect of drug cannot be ruled out. A clinical trial investigating the long-term effects of semaglutide in diabetic eye disease is ongoing (NCT03811561).

In summary, DDP-4i, GLP-1RA, and SGLT-2i are generally well tolerated in T2DM patients, but the following items should be considered: when choosing GLP-1RA, attention should be paid to gastrointestinal adverse reactions (a gradual increase in the dose may help reduce symptoms); Simultaneous use of insulin or sulfonylureas will increase the risk of GLP-1RA hypoglycemia (should pay special attention to elderly patients); Similarly, when DDP-4i is used in combination with sulfonylureas, the dose of the latter should also be considered; Use GLP-1RA and DPP-4i with caution if there is a history of pancreatitis; GLP-1RA should be used with caution if there is a history of bile duct and gallbladder disease; Make sure to screen for retinopathy before starting (only for semaglutide); For personal or family history of medullary thyroid carcinoma or multiple endocrine tumors, use GLP-1RA with caution; The use of DDP-4i, SGLT-2i or GLP-1RA is prohibited during pregnancy and lactation; The use of SGLT-2i should be closely monitored for genital fungal infection (it is recommended to drink more water); Carefully monitor the lower extremity ulcers. If the patient develops lower extremity complications, consider stopping treatment (at least suspend use until symptoms are relieved).

It should be noted that we listed most of the risks of the new hypoglycemic drugs, but there may be other risks that have not been discussed.

## 3 Discussion and Future Perspectives

### 3.1 Initial Combination or Traditional Stepwise Therapy in Prediabetes or T2DM

Earlier studies supported initial combination therapy to achieve glycemic goals more rapidly while reducing the incidence of hypoglycemia (compared to traditional stepwise therapy) (Phung et al., 2014; Abdul-Ghani et al., 2015). Recently, the VERIFY trial randomly assigned 2001 eligible T2DM patients [such as body-mass index (BMI), 22–40 kg/m^2^ and HbA_1c_, 6.5–7.5%] to the early stage combination therapy group [50 mg vildagliptin (DPP-4i) twice a day + metformin] or initial metformin monotherapy group (stable daily dose of 1, 1.5 or 2 g). The 5-years treatment period is divided into 3 study periods. In period 1, if the initial monotherapy did not maintain HbA_1c_ < 7.0%, the monotherapy was replaced with a combination treatment. Entering period 2, all patients received vildagliptin + metformin treatment. Finally, 1,598 (79.9%) patients completed the 5 years study, and the numbers in the early combination treatment group and monotherapy group were 811 (81.3%) and 787 (78.5%), respectively. In period 1, the number of initial treatment failures in the combination treatment group and monotherapy group was 429 (43.6%) and 614 (62.1%), respectively. The median time to medication failure observed in the two groups was 61.9 months (estimated) and 36.1 months, respectively. During the 5 years treatment period, compared with metformin monotherapy, the relative risk of initial treatment failure in vildagliptin + metformin combination therapy was obviously lower (HR 0.51; 0.45–0.58). In addition, both treatments are safe and well tolerated. These results indicate that for newly diagnosed T2DM patients, the early use of vildagliptin combined with metformin will provide greater, longer-lasting long-term benefits compared to traditional stepwise therapy (Figure 1C) (Matthews et al., 2019b) (Table 3).

**TABLE 3:** Randomized controlled trial of early combination therapy with new hypoglycemic drugs in T2DM and prediabetes.

	Groups	Duration (wks)	*n*	Inclusion Criteria	HbA1c changes (%)	Weight Changes (kg)
**Early Combination of SGLT-2i and Metformin**						
NCT01719003 Hadjadj et al. (2016a) 2016	Empagliflozin + Metformin, both twice daily (12.5 mg + 1 g, 12.5 mg + 0.5 g, 5 mg + 1 g, 5 mg + 0.5 g) vs. Empagliflozin once daily (25 or 10 mg) vs. Metformin twice daily (1 or 0.5 g)	24	1,364	T2DM, Baseline age (years): 50.3–53.6, patients who were drug-naïve (no OAM treatment, insulin, or GLP-1 analog for ≥12 weeks)	(−2.08, −1.93, −2.07, −1.98) vs. (−1.36, −1.35) vs. (−1.75, −1.18)	(−3.8, −3.0, −3.5, −2.8) vs. (−2.4, −2.4) vs. (−1.3, −0.5)
				Baseline HbA1c (%): 8.58–8.86	HbA1c < 7.0% (68%, 57%, 70%, 63%) vs. (32%, 43%) vs. (58%, 38%)	Weight loss >5%: (40.8%, 29.7%, 36.5%, 26.1%) vs. (28.0%, 24.9%) vs. (12.8%, 6.5%)
				Baseline BMI (kg/m^2^): 30.1–30.6		
NCT01809327 Rosenstock et al. (2016) 2016	Canagliflozin (100 or 300 mg) + Metformin, vs. Canagliflozin (100 or 300 mg) vs. Metformin	26	1,186	Drug-naïve T2DM, aged 18–75 years, HbA1c 7.5∼12%	(−1.77, −1.78) vs. (−1.37, −1.42) vs. −1.30	(−3.2, −3.9) vs. (−2.8, −3.7) vs. −1.9
				Baseline HbA1c (%): 8.8 (1.2)	HbA1c<7.0%: (49.6%, 56.8%), (38.8%, 42.8%) vs. 43.0%	
				Baseline BMI (kg/m^2^): 32.5 (5.8)		
**Early combination of DPP-4i and Metformin**						
PRELLIM Guardado-Mendoza et al. (2020) 2020	Linagliptin 5 mg + Metformin 1.7 g daily + lifestyle vs. Metformin 1.7 g daily + lifestyle	96	144	Patients with IGT plus two T2DM risk factors according to ADA, age between 18 and 65 years	Incidence of T2DM: 10 cases vs. 35 cases	−4.3 vs. −4.1
				Baseline HbA1c (%): 5.4–5.8		
				Baseline BMI (kg/m^2^): 28.1–30.5		
VERIFY Matthews et al. (2019a) 2019	Metformin (stable daily dose of 1, 1.5, or 2 g) + Vildagliptin 50 mg twice daily vs. Metformin (stable daily dose of 1, 1.5, or 2 g)	240	2001	T2DM patients (diagnosed within 2 years), aged 18–70 years old, HbA1c 6.5–7.5%	Initial treatment failure during period 1: 429 (43.6%) vs. 614 (62.1%)	Slight reduce in weight was apparent in both groups
				Baseline HbA1c (%): 6.7 (0.4–0.5)	The median time to treatment failure 61.9months (estimated) vs. 36.1month (observed)	
				Baseline BMI (kg/m^2^): 31.0–31.2		
NCT00382096 and NCT00468039 Bosi et al. (2009) 2009	Vildagliptin 50 mg + Metformin (1 g, 0.5 mg) twice daily vs. Vildagliptin 50 mg or Metformin 1 g twice daily	24	1,179	Treatment-naive patients with T2DM who aged 18–78 years old	(−1.8%, −1.6%) vs. −1.1%, −1.4%	(−1.19, −1.17) vs. −1.62, 0.59
				Baseline HbA1c (%): 8.6–8.7	HbA1c < 7.0%: (65.4%, 55.4%) vs. 40.0%, 43.5%	
				Baseline BMI (kg/m^2^): 31.3 (21.1–44.1)		
NCT00103857 Williams-Herman et al. (2009) 2009	Sitagliptin 50 mg + Metformin (1 g, 0.5 g) twice daily vs. Metformin (1 g, 0.5 g) twice daily vs. Sitagliptin 100 mg once daily	54	1,091	T2DM patients, aged 18–78 years old	(−1.8, −1.4) vs. (−1.3, −1.0) vs. −0.8	(−1.7, −0.7) vs. (−1.5, −1.0) vs. −0.6
				Baseline HbA1c (%): 8.4–8.8	HbA1c < 7.0%: (67%, 48%) vs. (44%	
				Baseline BMI (kg/m^2^): 31–32	25%) vs. 23%	
NCT00482729 Olansky et al. (2011) 2011	Sitagliptin/Metformin 50/500 mg up to 50/1,000 mg vs. Metformin	44	1,250	T2DM patients, aged 18–78 years old	−2.3% vs. −1.8%	−1.1 vs. −1.2
	500 mg up to 1,000 mg twice-daily			Baseline HbA1c (%): 9.8–9.9 (1.8)	HbA1c < 7.0%	
				Baseline BMI (kg/m^2^): 22.9–25.3	46.1% vs. 30.4	
NCT00327015 Pfützner et al. (2011) 2011	Saxagliptin (5 mg, 10 mg), once daily + Metformin 0.5 g, twice daily vs. Saxagliptin	76	1,306	Treatment-naive patients with T2DM who aged 18–77 years old	(−2.31%, −2.33%) vs.−1.55% vs.−1.79%	(−1.2, −0.7) vs. −0.3 vs. −1.0
	10 mg vs. Metformin 0.5 g			Baseline HbA1c (%): 9.4–9.6	HbA1c<7.0%: (51.1%, 50.8%) vs.	
				Baseline BMI (kg/m^2^): 29.9–30.4	25 vs. 34.7%	
**Early combination of GLP-1RA/metformin/pioglitazone or metformin/sulfonylurea/insulin**						
EDICT Abdul-Ghani et al. (2015) 2015	Metformin + Pioglitazone + Exenatide (triple therapy) vs. Sequential add-on therapy with Metformin, Sulfonylurea and then Basal Insulin	104	249	T2DM patients (diagnosed within 2 years), aged 30–75 years old	HbA1c < 6.0%: 61 vs. 27%	−1.2 vs. +4.1
				Baseline HbA1c (%):8.6 (0.2)		
				Baseline BMI (kg/m^2^): 36.4–36.6	7.5-fold lower rate of hypoglycaemia vs. Sequential add-on	

Abbreviations: ADA, the American Diabetes Association; BMI, body-mass index; DPP-4i, dipeptidyl peptidase-4 inhibitors; GLP-1RA, glucagon-like peptide 1 receptor agonists; HbA1c, glycated haemoglobin; MTD, maximum tolerated dose; OAM, oral antihyperglycemic medication; SGLT-2i, sodium-glucose cotransporter 2 inhibitors.

Moreover, the research results of Hadjadj et al. (2016b) showed that among T2DM patients who were not treated with hypoglycemic drugs, empagliflozin + metformin, both twice daily (5 mg + 1 g) were close to (12.5 mg + 1 g) in reducing HbA_1c_. And it is significantly better than once daily empagliflozin (25 mg) or twice daily metformin (1 g) treatment. The mean HbA_1c_ (%) decrease from baseline was −2.07, −2.08, −1.36, and −1.75, respectively. And the mean weight loss from baseline was −3.5, −3.8, −2.4, and −1.3 kg, respectively. This suggests that low-dose empagliflozin and high-dose metformin combined therapy is superior to high-dose empagliflozin or metformin in controlling blood glucose and weight. The study by Rosenstock et al. (2016) (NCT01809327) also had similar results: it is recommended to initiate canagliflozin + metformin combination therapy in T2DM patients who did not receive hypoglycemic drugs (Figure 1C).

Prediabetes is a very common health problem that progresses to T2DM and the risk of T2DM complications are high. Lifestyle interventions (a combination of diet and exercise aimed at reducing weight and increasing activity levels) can improve glucose tolerance and prevent progression from impaired glucose tolerance (IGT) to T2DM. Considering the reduction in morbidity in the short term, as well as the modest benefit in terms of side effects and/or lack of proven cardiovascular benefit. On this basis, the FDA and EMA in the United States and Europe announced that there are no drugs recommended for T2DM prevention. Nevertheless, there may still be value in combination therapy with new hypoglycemic agents for prediabetes.

The PRELLIM trial randomly divided 144 pre-diabetic patients into 5 mg linagliptin (DPP-4i) + metformin 1.7 g + lifestyle (LM group) or metformin 1.7 g + lifestyle (M group) to evaluate the incidence of linagliptin on T2DM and other related events. The results indicated that glucose levels during oral glucose tolerance test (OGTT) in the LM group were significantly improved. The OGTT disposition index (DI) of the LM group increased from 1.31 to 2.41 (6 months) and 2.07 (24 months), while the DI of the M group increased from 1.21 to 1.56 (6 months) and 1.72 (24 months). In addition, the LM group is more likely to reach normal blood glucose [Odds Ratio (OR) 3.26; 1.55–6.84], while the M group is more likely to develop T2DM (HR 4.0; 1.24–13.04). No obvious adverse reactions were observed during the study. These findings indicate that in patients with prediabetes, linagliptin + metformin + lifestyle can obviously improve pancreatic β cell function, glucose metabolism, and reduce the incidence of T2DM (Guardado-Mendoza et al., 2020). Similarly, compared with prediabetic patients who treated with placebo, dapagliflozin also decreased the risk of new-onset T2DM by 32%, which is similar to the metformin effect observed in diabetes prevention studies (approximately 31%) (Brown et al., 2021). This suggests that in patients with prediabetes, the early combination of SGLT-2i and metformin may further reduce the incidence of T2DM (Figure 1A).

Research by Yusuf et al. (2021) showed that polypill (with or without aspirin) containing different antihypertensive drugs and statins provides long-term benefits for participants who do not have CVD but are at moderate cardiovascular risk (average follow-up time is 4.6 years, polypill reduces the occurrence of cardiovascular events). The research results of Chow et al. (2017) showed that quadpill treatment (contains four antihypertensive drugs, each quarter dose: amlodipine 1.25 mg, atenolol 12.5 mg, hydrochlorothiazide 6.25 mg, and irbesartan 37.5 mg) has a good blood pressure control effect for participants with untreated hypertension. The benefits of polypill and quadpill in participants with moderate CV risk or initial antihypertensive therapy (reducing the dose of the drug may help reduce adverse reactions and cancel the requirement for dose adjustment), which has important reference value for the combination therapy of hypoglycemic drugs (increase patient compliance, reduce the failure rate of single-drug hypoglycemic, reduce single-drug dose to control costs).

The latest data (IDF diabetes atlas 10th edition) shows that 541 million adults worldwide have IGT. It is particularly important to effectively reduce their risk of developing T2DM. More than three-quarters of adults with diabetes live in countries with low and moderate incomes. The medical expenditure caused by diabetes is at least US$966 billion (increased by 316% in the past 15 years) (IDFdiabetes atlas 10th edition). Therefore, it is also very important to control the cost of diabetes treatment. Undoubtedly, as the new hypoglycemic drugs become widely available and their patents expire, their prices will be greatly reduced. On the other hand, adjusting the dosage of hypoglycemic drugs and the initial combination therapy (both in prediabetes and newly diagnosed T2DM patients) may help improve efficacy and reduce costs (including reducing the cost of expensive drugs and the medical burden due to treatment failure).

In fact, DPP-4i and metformin, and the combination of SGLT-2i and metformin have been approved in some countries and regions (such as the European Union and Japan), but are mainly used in patients with poor monotherapy or those who are taking the drugs in the combination separately (eg empagliflozin and metformin hydrochloride). Therefore, studies of approved or unapproved combination drugs for the initial treatment of prediabetes and T2DM should be accelerated. The approval of oral GLP-1RA (semaglutide) will help to promote this process.

### 3.2 Cardioprotective Mechanism of SGLT-2i

**FIGURE 2:**
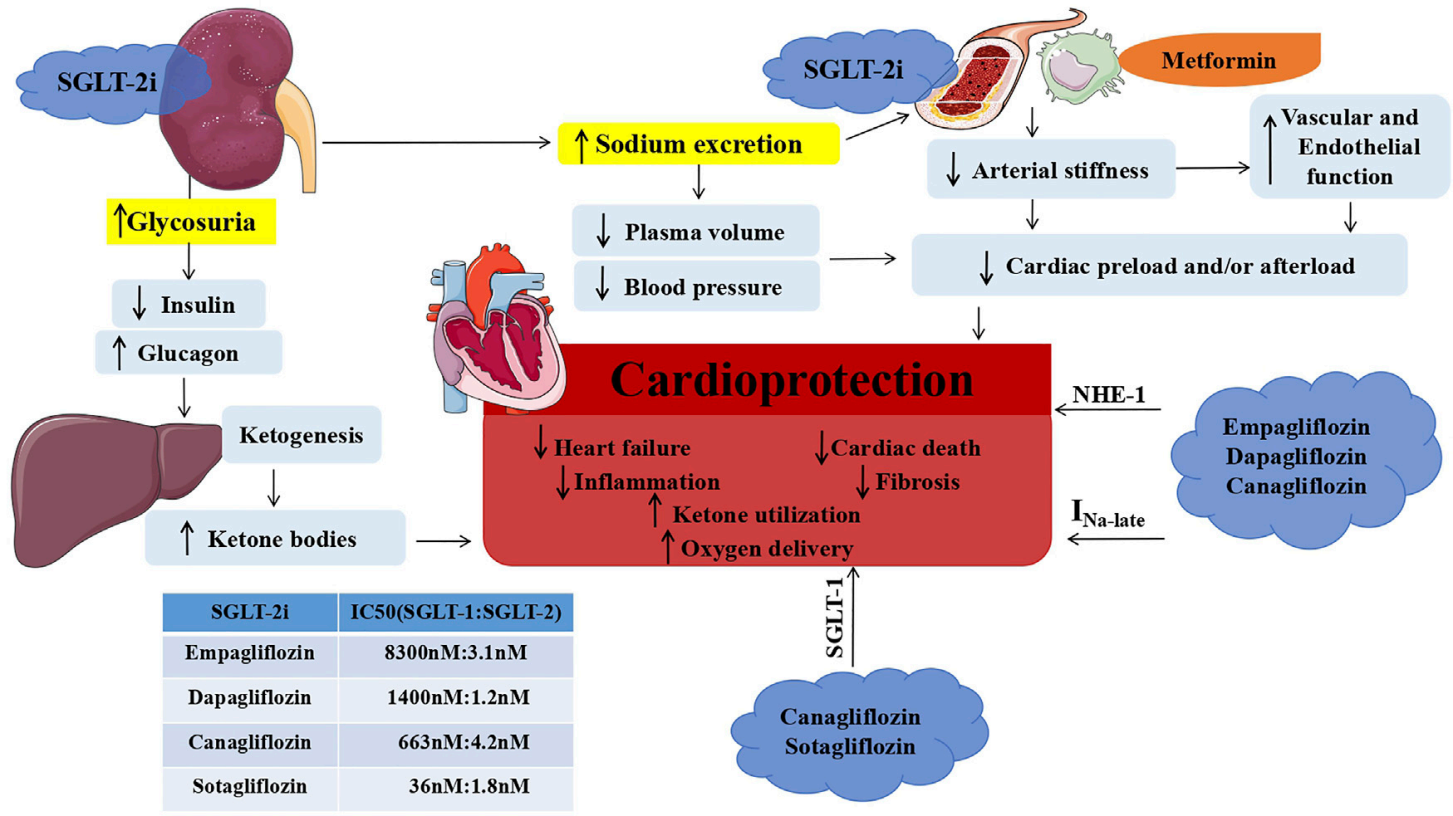
The mechanism of SGLT-2i in cardioprotection. The comprehensive cardioprotective mechanism of SGLT-2i (including indirect and direct effects): SGLT-2i promotes urinary glucose excretion by inhibiting SGLT-2 on the kidneys, lowers insulin, increases glucagon, and then promotes the increase of ketone bodies through the ketogenic effect of the liver; In addition, SGLT-2i increases urinary sodium excretion, reduces blood volume and blood pressure, and reduces preload and afterload of the heart. These indirect effects on vascular cells (including endothelial cells and macrophages) are shown to reduce arterial stiffness and improve vascular and endothelial functions. Metformin can increase the beneficial effects of SGLT-2i on atherosclerosis. The direct effects of SGLT-2i include empagliflozin, dapagliflozin, and canagliflozin to inhibit NHE-1 and late Na⁺ in cardiomyocytes. At the same time, canagliflozin inhibits cardiac SGLT-1. Sotagliflozin, a non-selective SGLT2i, is speculated to have this effect. The above-mentioned indirect and direct cardioprotective effects have improved heart function and decreased the rate of hospitalization for HF and cardiovascular mortality. Abbreviations: NHE1, Na^+^/H^+^ exchanger 1; SGLT-1, sodium-glucose cotransporter 1; SGLT-2i, sodium-glucose cotransporter 2 inhibitors.

In general, the cardioprotective mechanism of SGLT-2i is mainly divided into two parts: indirect and direct effects (Figure 2): indirect effects mainly include lowering blood pressure and weight, ketogenesis, and lowering glucose. The results of DAPA-HF, EMPEROR-Reduced and EMPEROR-Preserved indicate that SGLT-2i has similar benefits in diabetic and non-diabetic patients, suggesting that the protective effect of SGLT-2i on HF may be independent of its hypoglycemic effect. However, SGLT-2i decreases the risk of conversion to T2DM in the prediabetes stage (Brown et al., 2021), indicates that SGLT-2i is beneficial to the blood glucose management of non-diabetic patients. As we all know, the SGLT-2i hypoglycemic pathway is significantly different from other hypoglycemic drugs. Whether this special glucose-lowering pathway (improving energy balance) is beneficial for HF remains unclear. Therefore, the protective mechanism of SGLT-2i on HF in non-diabetic patients cannot be completely ruled out for its hypoglycemic effect. The direct effects include Na^+^/H^+^ exchanger 1 (NHE1) inhibition and late Na⁺ inhibition (empagliflozin, dapagliflozin, and canagliflozin) (Uthman et al., 2018; Philippaert et al., 2021) as well as SGLT1 inhibition [canagliflozin (Schiattarella and Bode, 2021). Sotagliflozin as a non-selective SGLT-2i should have similar effects]. Although SGLT-2i have obvious protective effects against HF, their direct cardioprotective mechanism may be different.

Dynamic changes in gene expression can lead to progressive organ dysfunction (including HF) (Alexanian et al., 2021). The cardioprotective effect of SGLT-2i should be a comprehensive effect of its indirect and direct effects. How such a comprehensive effect regulates the expression of genes in the heart (cardiomyocytes and non-cardiomyocytes) to improve heart function is still unclear. The use of single-cell sequencing (such as scRNA-seq and scATAC-seq) will help the study of the comprehensive mechanism of SGLT-2i′s cardioprotection. The key pathways of HF that SGLT-2i cannot regulate may be an important research direction for HF drug treatment in the future.

A lot of research has been devoted to discovering new potential disease targets, but it is unknown whether these targets can be intervened by existing clinical drugs. Therefore, we discussed the cardioprotective mechanism of SGLT-2i, and provided ideas for the development of HF drugs from the perspective of “clinical drug-mechanism-intensive disease treatment.” This will help accelerate the development of HF drugs. Through the analysis of the signaling pathways of different therapeutic drugs, it may also guide the combined application of drugs to a certain extent.

## 4 Conclusion

New hypoglycemic drugs are generally well tolerated, although caution is required in rare cases. The research and development of compound hypoglycemic drugs (especially containing SGLT-2i and/or oral GLP-1RA) should be carried out in prediabetes and newly diagnosed T2DM as soon as possible. On this basis, we further provide research ideas for the treatment of HF complications in T2DM. These insights will aid in the management of blood glucose and risk of complications (especially HF) in patients with prediabetes and T2DM.
