# Supplementary material for: New Hypoglycemic Drugs: Combination Drugs and Targets Discovery
Source: Front Pharmacol. 2022 Jun 8;13:877797. doi: 10.3389/fphar.2022.877797 (PMC9295075; doi:10.3389/fphar.2022.877797)
Supplement: Supplementary file 1 [file Table1.docx]

| **Table S1. Randomized controlled trials of GLP-1RA in T2DM (mainly phase 3)** | | | | | | | | | | | | | |
| --- | --- | --- | --- | --- | --- | --- | --- | --- | --- | --- | --- | --- | --- |
|  | **Groups** | **Duration (wks)** | | | | **n** | | **Inclusion criteria** | | **HbA_1c_**  **changes (%)** | | | **Weight changes (kg)** |
| **GLP-1RA** | | | | | | | | | | | | | |
| **Liraglutide *vs.* placebo or other GLP-1RA** | | | | | | | | | | | | | |
| Ellipse ^(^[^Tamborlane^ *^et al.^*^, 2019^](#_ENREF_19)^)^  2019 | Liraglutide  1.8 mg/d  *vs.* Placebo | 52 | | | | 135 | | T2DM, 10~17 years old, HbA_1c_ 7.0~11.0% with diet and exercise alone, HbA1c 6.5~11.0% with metformin (with or without insulin)  Baseline HbA1c (%): 7.78 (1.34)  Baseline BMI (kg/m^2^): 33.9(9.25) | | HbA_1c_ difference：  -1.06% (26 wks)  -1.30% (52 wks)  HbA_1c_<7.0%:  63.7% *vs.* 36.5% | | | -2.3 *vs.* -0.99  (26 wks)  -1.91 *vs.* 0.87  (52 wks) |
| LEAD-6 ([Buse *et al.*, 2009](#_ENREF_5)) 2009 | Liraglutide 1.8 mg/d *vs.* Exenatide 10 μg twice per day | 26 | | | | 464 | | T2DM, 18 to 80 years old, HbA_1c_ 7.1~11.0%, MTD of metformin, sulfonylureas, or both.  Baseline HbA_1c_ (%): 8.1~8.2  Baseline BMI (kg/m^2^): 32.9 (5.5~5.7) | | -1.12 *vs.*-0.79  HbA_1c_<7.0%:  54% *vs.* 43% | | | -3.24 *vs.*-2.87 |
| DURATION-6 ([Buse *et al.*, 2013](#_ENREF_4))  2013 | Liraglutide 1.8 mg/d  *vs.* Exenatide 2 mg once per week | 26 | | | | 912 | | T2DM, 18 years or older, HbA_1c_ 7.1~11.0%, receiving OAM (sulfonylurea, metformin, or both of them, or metformin + pioglitazone).  Baseline HbA_1c_ (%): 8.4~8.5  Baseline BMI (kg/m2): 32.3 (5.4~5.6) | | -1.48 *vs.* -1.28  HbA_1c_<7.0%：  60% *vs.* 53% | | | -3.57 *vs.*-2.68 |
| NCT01973231 ([Nauck *et al.*, 2016](#_ENREF_13))  2016 | Liraglutide 1.8 mg/d *vs.* Lixisenatide 20 µg/d | 26 | | | | 404 | | T2DM, aged ≥18 years, HbA_1c_ 7.5~10.5%, with metformin therapy at the MTD (1 to 3 g/d) for ≥90 days.  Baseline HbA_1c_ (%): 8.4 (0.7~0.8)  Baseline BMI (kg/m^2^): 34.5~34.9 | | -1.8 *vs.* -1.2  HbA_1c_<7.0%：  74.2% *vs.* 45.5% | | | -4.3 *vs.*-3.7 |
| HARMONY 7 ([Pratley *et al.*, 2014](#_ENREF_16))  2014 | Liraglutide 1.8 mg/d *vs.* Albiglutide 50 mg once per week | | 32 | | | 841 | T2DM, aged ≥18 years, HbA_1c_ 7.0~10.0%, receiving metformin, sulfonylureas, thiazolidinediones, or any combination of them.  Baseline HbA_1c_ (%): 8.15~8.18  Baseline BMI (kg/m^2^): 32.8 (5.9~6.0) | | | -0.99 *vs.*-0.78  HbA1c <7.0%:  52% *vs.* 42% | | | -2.16 *vs.*-0.64 |
| AWARD-6 ([Dungan *et al.*, 2014](#_ENREF_9))  2014 | Liraglutide 1.8 mg/d *vs.* Dulaglutide 1.5 mg once weekly | 26 | | | | 599 | | T2DM, aged ≥18 years, HbA_1c_ 7.0~10.0%, with metformin (≥1.5g/day) therapy.  Baseline HbA_1c_ (%): 8.1 (0.8)  Baseline BMI (kg/m^2^): 33.5~33.6 | | -1.36 *vs.*-1.42  HbA_1c_<7.0%：  68% *vs.* 68% | | | -3.61 *vs.*-2.90 |
| PIONEER 4 ([Pratley *et al.*, 2019](#_ENREF_14))  2019 | Liraglutide 1.8 mg/d *vs.* Semaglutide (oral) 14 mg/d *vs.* Placebo | 52 | | | | 711 | | T2DM, aged ≥18 years,  HbA_1c_ 7.0~9.5%, treatment with daily dose of metformin (≥1.5g or MTD) with or without SGLT-2i.  Baseline HbA_1c_ (%): 8.0 (0.7)  Baseline BMI (kg/m^2^): 33.0 (6.3) | | -0.9 *vs.* -1.2 *vs.* -0.2  HbA_1c_<7.0%:  55.0% *vs.* 60.7% *vs.* 15% | | -3.0 *vs.* -4.3 *vs.*-1.0  Weight loss≥5%: 24.5% *vs.* 44.7% *vs.* 12.0% | |
| **Semaglutide (comparison between different preparations and doses),**  **or *vs.* placebo or other GLP-1RA or insulin** | | | | | | | | | | | | | |
| SUSTAIN 3 ([Sorli *et al.*, 2017](#_ENREF_18))  2017 | Semaglutide 0.5 mg, 1.0 mg, once-weekly  *vs.* Placebo | | | | 30 | 387 | T2DM, aged ≥18 years, HbA_1c_ 7.0~10.0%, diet and exercise therapy alone for ≥30 days.  Baseline HbA_1c_ (%): 8.05 (0.85)  Baseline BMI (kg/m^2^): 32.93 (7.68) | | | | -1.45, -1.55 *vs.* -0.02  HbA_1c_<7.0%：  74%, 72% *vs*. 25% | | -3.73, -4.53 *vs.*-0.98 |
| PIONEER 8 ([Zinman *et al.*, 2019](#_ENREF_22))  2019 | Semaglutide  3, 7 or 14 mg/d oral *vs.* Placebo | | | | 52 | 731 | Adult patients with T2DM, HbA_1c_ 7.0~9.5%, stable insulin therapy (≥10 units/day) for ≥3 months.  If metformin is used, a stable daily dose (≥1.5g or MTD) for ≥90 days is required.  Baseline HbA_1c_(%): 8.2 (0.7)  Baseline BMI (kg/m^2^): 31.0 (6.7) | | | | -0.6, -0.9, -1.3 *vs.* -0.1  HbA_1c_<7.0%: 28.9%, 39.6%, 54.2% *vs.* 9.3% | | -1.4, -2.4, -3.7 *vs.* -0.4  Weight loss ≥5%: 17.2%, 28.1%, 39.1% *vs.* 5.2% |
| SUSTAIN FORTE ([Frías *et al.*, 2021a](#_ENREF_10))  2021 | Semaglutide  2.0 mg *vs.* 1.0 mg, both once-weekly | | | | 40 | 961 | T2DM, aged ≥18 years, HbA1c 8.0~10.0%, taking a stable metformin therapy (≥1.5g/d or MTD) alone or in combination with a sulfonylurea for ≥90 days.  Baseline HbA_1c_ (%): 8.9 (0.6)  Baseline BMI (kg/m^2^): 34.6 (7.0) | | | | -2.2 *vs.* -1.9  HbA_1c_<7.0%:  57.5%*vs.* 67.6% | | -6.9 *vs.* -6.0 |
| NCT01923181 ([Davies *et al.*, 2017](#_ENREF_7))  2017 | Semaglutide of 2.5, 5, 10, 20, 40 mg/d, oral *vs.* placebo *vs.* Semaglutide 1.0 mg subcutaneous once per week | | | | 31 | 632 | T2DM, aged ≥18 years, HbA_1c_ 7.0~9.5%, diet and exercise therapy alone or metformin treatment≥30 days.  Baseline HbA_1c_ (%): 7.8~8.0  Baseline BMI (kg/m^2^): 30.7~32.6 | | | | -0.7, -1.2, -1.5, -1.7, -1.9 *vs.* -0.3, -1.9  HbA_1c_<7.0%: 44%, 81%, 84%, 86%, 90% *vs.* 28%, 93% | | -2.1, -2.7, -4.8, -6.1, -6.9 *vs.* -1.2, -6.4 |
| SUSTAIN 3 ([Ahmann *et al.*, 2018](#_ENREF_1))  2018 | Semaglutide 1.0 mg *vs.* Exenatide 2 mg, both once weekly | | | | 56 | 813 | T2DM, age ≥ 18 years old, HbA_1c_ 7.0~10.5%, treated with one or two OAM (metformin ≥1.5g/d or MTD, and/or sulfonylureas, and/or thiazolidinediones).  Baseline HbA_1c_ (%): 8.3 (6.5~11.2)  Baseline BMI (kg/m^2^): 33.8 (21.1~72.8) | | | | -1.5 *vs.* -0.9  HbA_1c_<7.0%：  67% *vs.* 40% | | -5.6 *vs.* -1.9 |
| SURPASS-2 ([Frías *et al.*, 2021b](#_ENREF_11))  2021 | Semaglutide 1.0 mg, *vs.* Tirzepatide  5 mg, 10 mg, 15 mg  subcutaneous  all once-weekly | | | | 40 | 1879 | T2DM, age ≥ 18 years old, HbA_1c_ 7.0~10.5%, with metformin (≥1.5g/d) therapy.  Baseline HbA_1c_ (%):8.28 (1.03)  Baseline BMI (kg/m^2^): 34.2 (6.93) | | | | -1.86 *vs.* -2.01, -2.24, -2.30  HbA_1c_<7.0%:  79% *vs.* 82%, 86%, 86% | | -5.7 *vs.* -7.6, -9.3, -11.2 |
| SUSTAIN 4 ([Aroda *et al.*, 2017](#_ENREF_2))  2017 | Semaglutide 0.5, 1.0 mg once per week *vs.* Insulin glargine (starting dose 10 IU/d) | | | | 30 | 1089 | T2DM, aged ≥18 years, HbA_1c_ 7.0~10.0%, insulin-naive and on therapy with metformin (or metformin + sulfonylurea) for ≥90 days.  Baseline HbA_1c_ (%): 8.2 (0.9)  Baseline BMI (kg/m^2^): 33.0 (6.5) | | | | -1.21, -1.64 *vs.* -0.83  HbA_1c_<7.0%：  57%, 73% *vs.* 38% | | -3.47, -5.17 *vs.* 1.15 |
| SUSTAIN 10 ([Capehorn *et al.*, 2020](#_ENREF_6))  2020 | Semaglutide 1.0 mg once per week *vs.* Liraglutide 1.2 mg/d | | | | 30 | 577 | T2DM, aged ≥18 years, HbA_1c_ 7.0~11.0%), treatment with the following OAM or combination: metformin (1.5 g or MTD), sulfonylurea or SGLT-2i.  Baseline HbA_1c_ (%): 8.2 (1.0)  Baseline BMI (kg/m^2^): 33.7 (6.8) | | | | -1.7 *vs.* -1.0  HbA_1c_<7.0%:  80% *vs.* 46% | | -5.8 *vs.* -1.9 |
| PIONEER 10 ([Yabe *et al.*, 2020](#_ENREF_21))  2020 | Semaglutide  once-daily oral 3, 7, or 14 mg/d *vs.* Dulaglutide 0.75 mg, once per week | | | | 52 | 458 | T2DM, aged ≥ 20 years, HbA_1c_ 7.0~10.5%, receiving a stable OAM monotherapy (sulfonylurea, glinide, alpha-glucosidase inhibitor, thiazolidinedione, or SGLT-2i) for ≥60 days.  Baseline HbA_1c_ (%): 8.3 (0.9)  Baseline BMI (kg/m^2^): 26.2 (4.8) | | | | -0.7, -1.4, -1.8 *vs.* -1.3  HbA_1c_<7.0%:  39%, 65%, 75% *vs.* 57% | | 0.1, -1.0, -1.9 *vs.* 1.1  Weight loss ≥5%:  5%, 17%, 25% *vs.* 7% |
| SUSTAIN 7 ([Pratley *et al.*, 2018](#_ENREF_15))  2018 | Semaglutide(0.5 mg, 1.0 mg) *vs.* Dulaglutide (0.75 mg, 1.5 mg),  all once weekly | | | | 40 | 1201 | T2DM, aged ≥18 years, HbA_1c_ 7.0~10.5%, treatment with daily dose of metformin monotherapy (1.5g or MTD).  Baseline HbA_1c_ (%): 8.2~8.3  Baseline BMI (kg/m^2^): 33.1~33.7 | | | | (-1.5, -1.8) *vs.* (-1.1, -1.4)  HbA_1c_<7.0%:  (68%, 79%) *vs.* (52%, 67%) | | (-4.6, -6.5) *vs.* (-2.3, -3.0) |
| **Dulaglutide (comparison between different doses), or *vs.* Exenatide** | | | | | | | | | | | | | |
| AWARD-11 ([Frias *et al.*, 2021](#_ENREF_12))  2021 | Dulaglutide 1.5, 3.0 *vs.*4.5 mg  once-weekly | | | 52 | | 1842 | | | T2DM, aged ≥18 years, HbA_1c_ 7.5~11.0%, with metformin (≥1.5g/d) therapy.  Baseline HbA_1c_ (%): 8.6(1.0)  Baseline BMI (kg/m^2^): 34.2(6.3) | -1.55, -1.61 *vs.* -1.72  HbA_1c_<7.0%：  49.3%, 54.1% *vs*. 59.4% | | | -3.4, -4.0 *vs.* -4.9 |
| AWARD-1 ([Wysham *et al.*, 2014](#_ENREF_20))  2014 | Dulaglutide 1.5mg, 0.75mg once per week *vs.* Exenatide 10 μg twice per day *vs.* placebo | | | 26 | | 976 | | | T2DM, 18 years or older, HbA_1c_ 7.0~11.0% on OAM monotherapy or HbA_1c_ 7.0~10.0%, on combination OAM therapy.  Baseline HbA_1c_ (%): 8.1 (1.2~1.3)  Baseline BMI (kg/m^2^): 33~34 | -1.51, -1.30 *vs.* -0.99 *vs.* -0.46  HbA_1c_<7.0%：  78%, 66% *vs.* 52% *vs.* 43% | | | -1.30, 0.20 *vs.* -1.07 *vs.* 1.24 |
| **Exenatide (weekly*vs.* daily injections), or *vs.* Lixisenatide** | | | | | | | | | | | | | |
| DURATION-1([Drucker *et al.*, 2008](#_ENREF_8))  2008 | Exenatide 2 mg once-weekly *vs.*  Exenatide 10 μg twice daily | | | 30 | | 295 | | | T2DM, 16 years or older, HbA_1c_ 7.1~11.0%,therapy with diet and exercise, or with 1~2 OAM (metformin, thiazolidinediones or sulfonylureas, or a combination)  Baseline HbA_1c_ (%): 8.3 (1.0)  Baseline BMI (kg/m^2^): 35(5) | -1.9 *vs.* -1.5  HbA_1c_ ≤7.0%:  77% *vs.* 61% | | | -3.7 *vs.* -3.6 |
| DURATION-5 ([Blevins *et al.*, 2011](#_ENREF_3))  2011 | Exenatide 2 mg once per week *vs.*  Exenatide 10 μg twice per day | | | 24 | | 254 | | | T2DM, 23~83 years old, no medication (19%) or one (47%) or multiple (35%) OAM treatments previously.  Baseline HbA_1c_ (%): 8.4~8.5  Baseline BMI (kg/m^2^): 33.0~33.6 | -1.6 *vs.* -0.9  HbA_1c_<7.0%：  58.1% *vs.* 30.1% | | | -2.3 *vs.* -1.4 |
| GetGoal-X  ([Rosenstock *et al.*, 2013](#_ENREF_17))  2013 | Exenatide 10 μg twice daily  *vs.* Lixisenatide 20 μg/d | | | 24 | | 634 | | | T2DM, aged 21~84 years, HbA_1c_ 7~10%, receiving ≥1.5 g/d metformin.  Baseline HbA_1c_ (%): 8.02 (0.8)  Baseline BMI (kg/m^2^): 33.6(6.4) | -0.96 *vs.* -0.79  HbA_1c_<7.0%：  49.8% *vs.* 48.5% | | | -3.98 *vs.* -2.96  Weight loss ≥5%: 31.4% *vs.* 25.1% |
| Abbreviation: ADA: the American Diabetes Association; BMI: body-mass index; DPP-4i: dipeptidyl peptidase-4 inhibitors; GLP-1RA: glucagon-like peptide 1 receptor agonists; HbA_1c_: glycated haemoglobin; MTD: maximum tolerated dose; OAM: oral antihyperglycemic medication; SGLT-2i: sodium-glucose cotransporter 2 inhibitors. | | | | | | | | | | | | | |
